# Posterior Oblique Approach for Sacroiliac Joint Fusion

**DOI:** 10.7759/cureus.33502

**Published:** 2023-01-08

**Authors:** Soubrata V Raikar, Thomas Nilles-Melchert, Arun A Patil, Walter Crum, Deepak Pandey

**Affiliations:** 1 Pain Management, Midwest Anesthesia Pain Management, Elkhorn, USA; 2 Surgery, Creighton University School of Medicine, Omaha, USA; 3 Ortho Spine, BNS consulting, Andover, USA

**Keywords:** percutaneous instrumentation, minimally-invasive spine, si joint fusion, chronic low back pain (clbp), sacroiliac joint dysfunctional pain

## Abstract

Introduction

Sacroiliac joint (SIJ) pain is a common source of low back pain. Though this condition can be treated with conservative measures, there is a subset of patients who fail to respond to conservative treatment. For them, surgical treatment using minimally invasive techniques could be considered. There are currently a number of SIJ fixation methods described. However, there is no case series reported on posterior oblique approach. Therefore, in this paper, the authors report their experience with the Sacrix system via the posterior oblique approach.

Method

In this series, 19 patients aged 44-84 years, with a median of 58 years, underwent SIJ fusion using this technique. This is a posterior oblique approach in which two screws are inserted into the ilium through the posterior part of the iliac crest and then advanced into the sacrum through the SIJ.

Results

The follow-up is between 7 and 30 months, with a median of 12 months. Eighteen patients had excellent pain relief. There was no complication from the procedure, and the blood loss was minimal. All eight patients who had follow-up radiographs showed solid fusion.

Conclusion

Posterior oblique approach for SIJ fusion is a minimally invasive procedure that proved to be effective and safe in this series. It also resulted in solid radiographic fusion, decreased pain, and improved function.

## Introduction

Sacroiliac joint (SIJ) pathology accounts for a significant portion of low back pain cases in the United States, with 15% to 30% of low back pain cases attributable to SIJ pathology [1,2]. Common etiologies include trauma, osteoarthritis, pregnancy, and surgical complications following lumbar spinal fusion, scoliosis, unequal lengths of the lower extremities, and less commonly seronegative arthropathies [3-5]. One review found that SIJ degeneration/arthrosis, SIJ dysfunction, postpartum instability, and SIJ trauma accounted for 59.8%, 18.4%, 7.2%, and 6.5% of SIJ pain cases, respectively [6].

Non-traumatic SIJ pain remains difficult to diagnose due to symptom overlap with other causes of low back pain such as discogenic or hip joint pain [7,8]. History and physical examination, while essential in establishing suspicion of SIJ pain, have limited reliability for diagnosis [8]. Specifically, there is a lack of specificity with SIJ pain provocation tests, which can be present in asymptomatic patients as well as other causes of low back pain such as sciatica [6]. The presence of three positive provocation tests has been reported to indicate a 65-93% probability of SIJ pain. Radiologic imaging is effective at excluding other causes but has a low sensitivity for SIJ pain, with one study showing that CT had a 57.5% sensitivity and 69% specificity [9]. Fluoroscopically guided SIJ block using local anesthetic and corticosteroids is the most common diagnostic modality for SIJ pain [10]. A pain relief cutoff of greater than 75% has been shown to be a good indication of SIJ joint pain [11]; however, no definitive diagnostic criteria have been established.

Surgical fusion of the SIJ as a treatment for SIJ-associated pain began in the early 1920s, with screws and plates introduced in the 1980s [4-6]. Due to high complication rates and long recovery, minimally invasive surgery procedures gained popularity beginning in 2008 and have now largely replaced the open technique [7,8]. Studies have demonstrated that SIJ fusion is a safe and effective treatment for SIJ pain with a low reoperation rate and high patient satisfaction, and is superior to non-surgical management [11-13].

As SIJ fusion has gained popularity, numerous techniques and devices have been employed, all of which mechanically stabilize the SIJ, with the ultimate goal of boney fusion of the joint. The SIJ can be approached laterally, posteriorly, or posterior obliquely, each with unique benefits and pitfalls [4-8]. The lateral approach needs dissection through the gluteal fascia, which is innervated by the cluneal nerves, then traverse the ileum before reaching the SIJ. The hardware is then implanted perpendicularly across the SIJ. The posterior approach involves minimal dissection and a more direct trajectory to the SIJ by avoiding the ileum laterally. However, it does have to cross the SIJ ligaments before reaching the articular surfaces. This approach allows implants to be inserted longitudinally through the joint. The posterior oblique approach described in this paper allows a direct trajectory to the SIJ through the ileum with the entry point on the outer upper surface of the iliac crest, avoiding gluteal dissection and SIJ ligaments.

Although there is a plethora of papers on SIJ fusion, there is no case series presentation on the posterior oblique approach using the Sacrix system in the literature. The authors, therefore are presenting this case series of 19 cases to add data to the literature on the Sacrix system.

This series is not comparing the Sacrix system to other devices. It is looking at one surgery center’s outcomes and the pearls and pitfalls of using this device. The results of this study were collected at one point in time resulting in various lengths of time post-operatively. This gives numerous data points post-operatively but creates heterogeneity when analyzing outcomes.

## Materials and methods

This case series consists of a single-center retrospective cohort analysis of 19 consecutive patients who underwent minimally invasive SIJ fusion using the Sacrix system via the posterior oblique approach in an outpatient setting. All patients who underwent the procedure from May 12, 2020, to April 13, 2022, were included in the study.

Patient characteristics

The age of the patients ranged from 44 to 84 years, with a median of 58 years. In total, 17 (89.5%) patients were female, 13 (68.4%) had right SIJ pain, 11 (57.9%) had a prior lumbosacral fusion, and one (5.3%) had a prior SIJ fusion using intra-articular screw. Eighteen (94.7%) patients received a prior SIJ injection with corticosteroids and local anesthetics, of which 16 (88.9%) reported more than 75% pain reduction. Thirteen (72.2%) patients showed bilateral degenerative joint disease affecting the SIJ on preoperative images.

Indication for surgery

Surgical candidacy was evaluated following failed conservative treatment including physical therapy, activity modification, and, in most cases, combined with corticosteroid and local anesthetic injection into the symptomatic joint. Indications for assessing candidacy included patient-reported history, positive provocation tests, failed conservative therapy, patients’ lifestyle, and preoperative safety assessment.

Data collection

All patients were assessed before the procedures and subsequently for pain level on a numerical rating scale ranging from 1 to 10. They were also assessed for their degree of functional improvement and their satisfaction with the procedure on a yes/no closed questionnaire.

The fusion system

**Figure 1:**
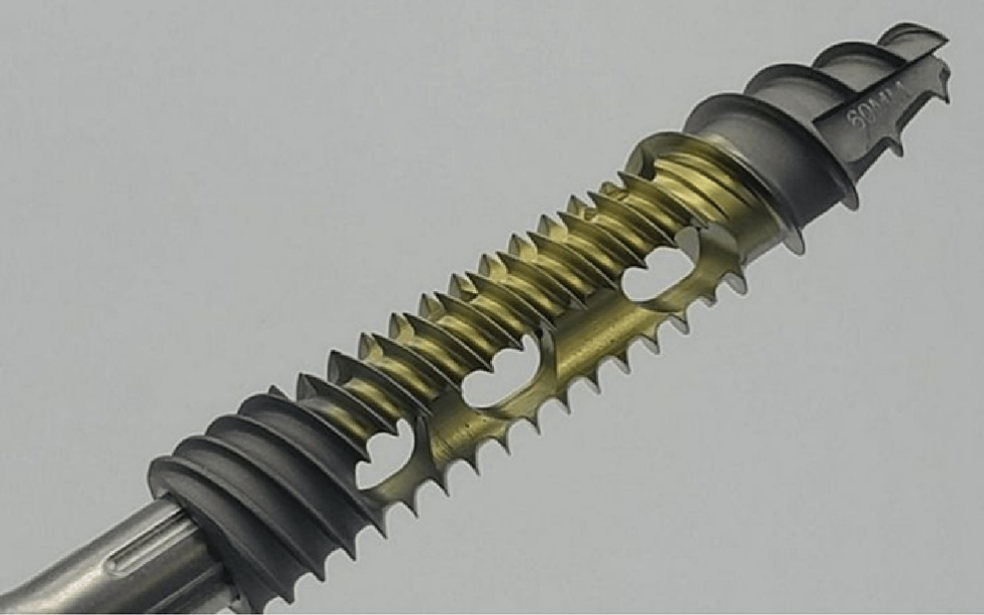
Photograph of a Sacrix screw

The Sacrix system (Figure 1) has self-tapping screws (manufactured by Sacrix, LLC, Malden, MA), with cortical and cancellous thread profiles, fusion channel for bone product delivery, and a bulleted tip. The implant sizes include diameters of 8, 10, 12, and 14 mm and lengths of 40, 45, 50, 55, and 60 mm. The most common size used in this series is a diameter of 12 mm and a length of 55 mm.

Surgical technique

**Figure 2:**
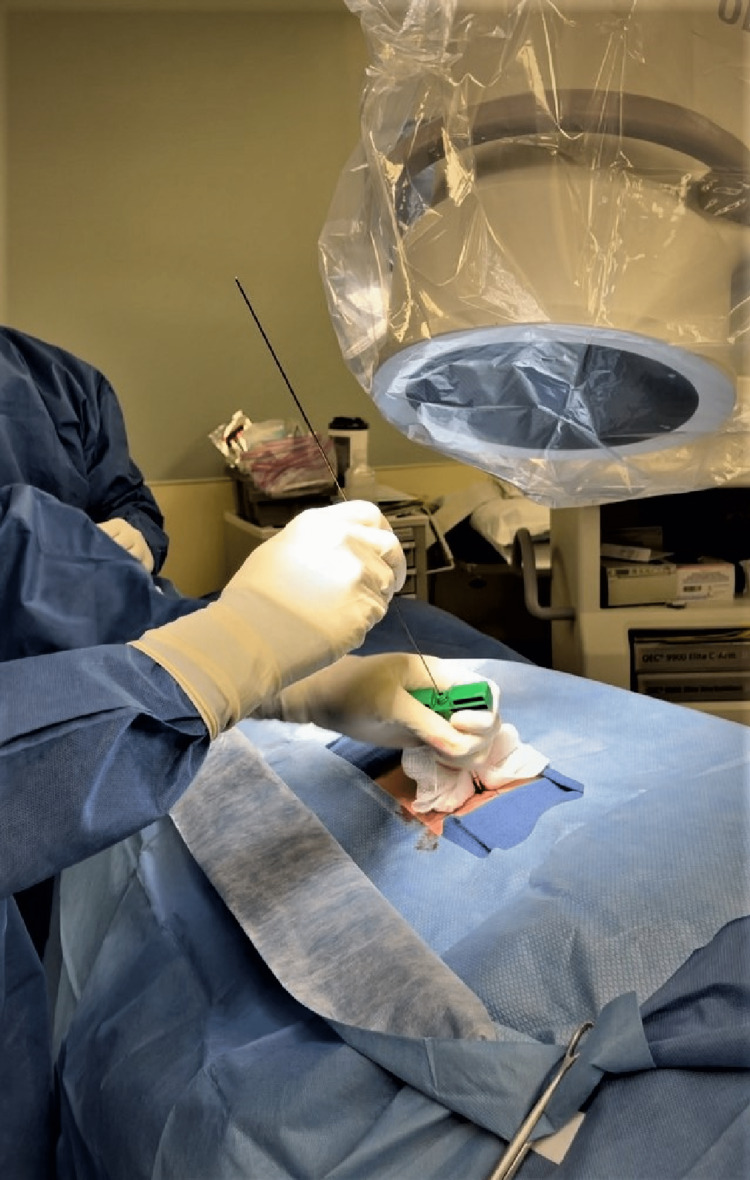
Photograph showing bone needle in the sacroiliac joint and insertion of the guide wire.

The procedure is carried out under general anesthesia using fluoroscopic guidance at every step. The patient is placed in a prone position. The SIJ and lateral aspect of the ileum are marked vertically, and the superior and inferior aspects of the sacral ala are marked horizontally, on the skin using fluoroscopic images. A bone needle tip is inserted with a trajectory approximately 5-20° to the horizontal at a point on the upper outer surface of the iliac crest at the level of the superior surface ala of the sacrum. The needle is advanced using a mallet through the ilium and SIJ to make it point to the anterior superior corner of the sacral ala. After confirming satisfactory needle placement, a guide wire is advanced (Figure 2), and the bone needle is removed.

**Figure 3:**
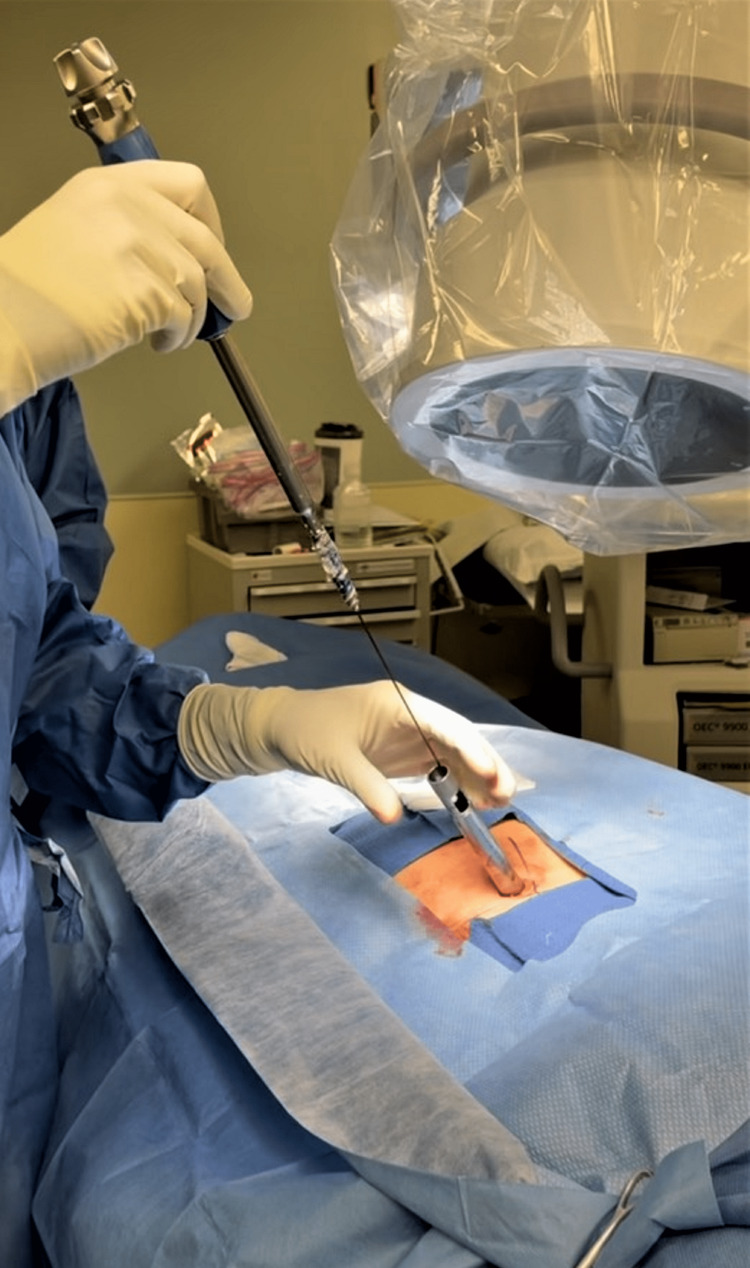
Photograph showing the screw, guided by the guide wire, being inserted into the sacroiliac joint through a tubular retractor.

**Figure 4:**
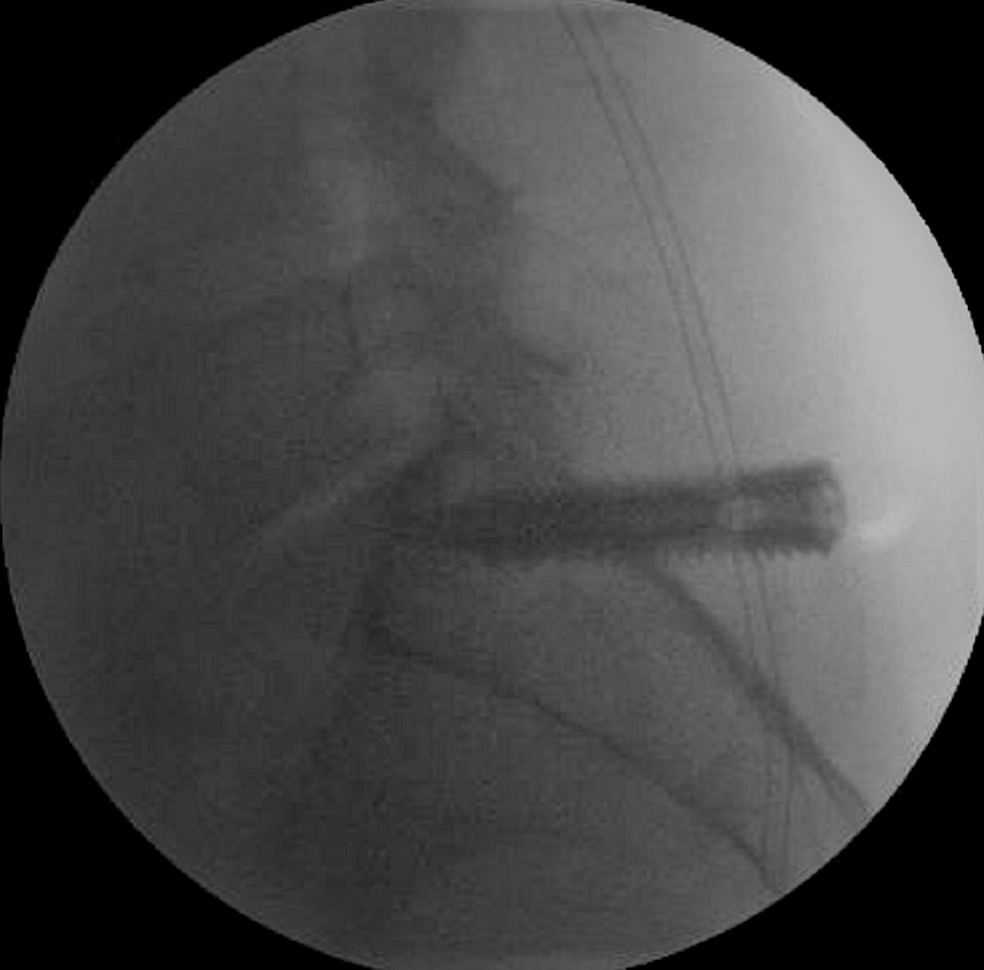
Lateral fluroscopic images showing the screw is aimed anteriorly upward with the tip just short of the superior surface of the ala of the sacrum.

A 1.5-cm incision is made longitudinally around the guide wire. A tissue dilator followed by a guiding cannula are then inserted. The tissue dilator is removed, and the cannula is then used for inserting the screw. A self-tapping screw imbedded with bone graft is then screwed in through the SIJ into the ala to its anterior superior surface (Figures 3, 4).

**Figure 5:**
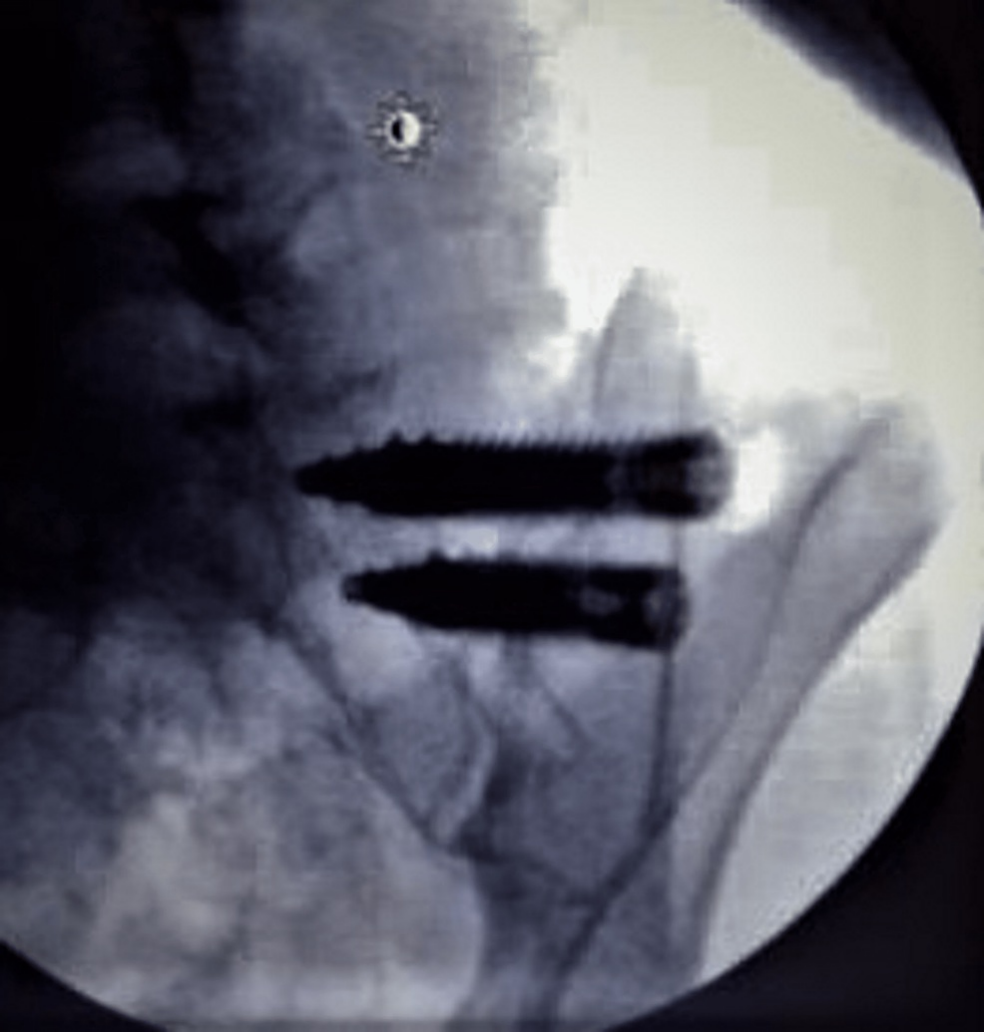
Intraoperative imaging study (patient 2) showing two screws inserted through the sacroiliac joint.

The second implant is inserted in a similar fashion approximately 1.5 cm caudal to the first implant through the same incision in a parallel trajectory (Figure 5). The incision is then closed. The patient is then discharged after successful recovery from anesthesia on the same day.

## Results

The follow-up is between 7 and 30 months, with a median of 12 months. There was no complication associated with these cases. There was minimal blood loss. Tables 1-3 show detailed results.

**Table 1:** Individual patient demographics and outcome SIJ, sacroiliac joint

Patient	Age	Sex	Follow-up in months	Side	Prior lumbar fusion	Positive response to SIJ injection	Pre-operative pain level	Post-operative pain level	% improvement in pain level	Satisfied
1	58	F	30	Right	Yes	Yes	8	3	62.5	Yes
2	47	F	30	Right	No	Yes	9	1	88	Yes
3	76	F	26	Right	No	No	8	0	100	Yes
4	58	F	26	Right	Yes	No	8	3	62.5	Yes
5	63	F	25	Right	No	Yes	9	2	77.7	Yes
6	46	F	23	Left	Yes	Yes	9	2	77.7	Yes
7	60	F	17	Left	Yes	Yes	10	4	60	Yes
8	81	M	15	Right	Yes	No injection	10	1	90	Yes
9	76	F	14	Right	No	Yes	9	2	77.7	Yes
10	82	F	12	Right	No	Yes	10	4	60	Yes
11	52	F	12	Right	No	Yes	10	3	70	Yes
12	47	F	12	Right	Yes	Yes	9	2	77.7	Yes
13	84	F	12	Left	Yes	Yes	10	0	100	Yes
14	60	F	11	Left	Yes	Yes	10	5	50	Yes
15	49	F	11	Right	Yes	Yes	8	3	62.5	Yes
16	68	F	9	Left	Yes	Yes	5	4	20	No
17	44	F	8	Right	No	Yes	9	1	88	Yes
18	59	M	8	Right	Yes	Yes	10	0.5	95	Yes
19	58	F	7	Left	No	Yes	9	1	88	Yes

**Table 2:** Summary of pre-operative care and imaging study results SI, sacroiliac

Patient characteristic	Percentage and numbers
Sex (female)	89% (17/19)
Laterality (right)	68.4% (13/19)
History of prior lumbar sacral fusion	57.9% (11/19)
History of prior SI fusion (intra-articular screw)	5.3% (1/19)
Failed conservative therapy	100% (19/19)
SI joint corticosteroid injection	94.7% (18/19)
Improvement with SI joint corticosteroid injection	88.9% (16/18)
Pre-operative imaging demonstrating degenerative joints	72.2% (13/18) 1 patient had unknown imaging history.

**Table 3:** Table shows patient assessment values

Patient assessment	Assessment values
Average pre-operative pain (1-10)	8.95 + 0.55 (95% CI: 8.4 to 9.5)
Average post-operative pain (1-10)	2.32 + 0.58 (95% CI: 1.74 to 2.9)
Average difference in pre-operative and post-operative pain scores	6.68 + 0.90 (95% CI: 5.78 to 7.58); P-value = 1.22 x 10^-11^
Patients with pain reduction	100% (19/19)
Patients with > 50% pain reduction	94.7% (18/19)
Satisfaction	94.7% (18/19)
Radiographic fusion after surgery	100% (8/8)
Functional improvement	94.7% (18/19)
Functional improvement; walking	84.2% (16/19)

Effectiveness of fusion in reducing pain

**Figure 6:**
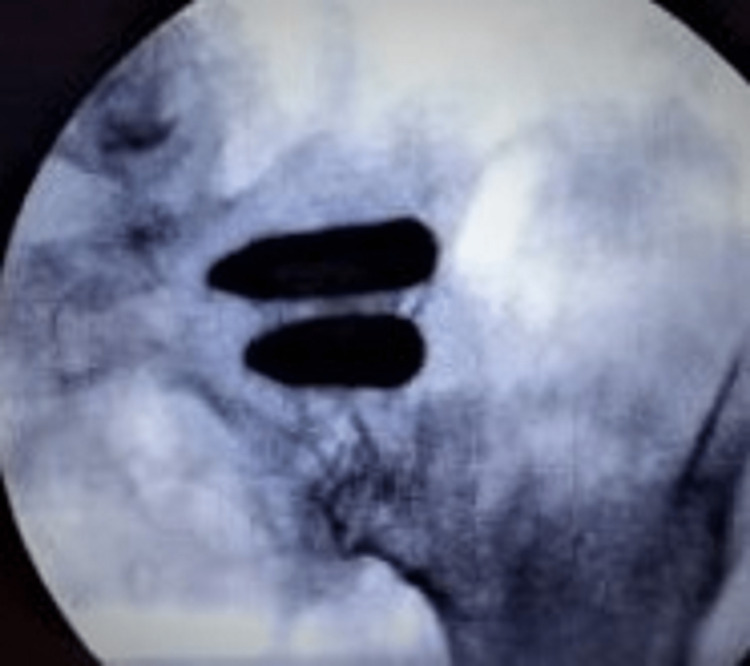
Radiographic image of the same patient (patient 2, as shown in Figure 4) 8 months after fusion. The implants look solid, without any empty spaces within them, and the screw threads are completely blunted indicating bony fusion.

All 19 (100%) patients in this case series had pain reduction; 18 (94.7%) patients had 50% or more reduction in their pain. Average preoperative pain score was 8.95 + 0.55 (95% CI: 8.4 to 9.5). Average postoperative pain score at the time of data collection was 2.32 + 0.58 (95% CI: 1.74 to 2.9) (Table 2). Average reduction in pain score was 6.68 + 0.90 (p-value = 1.22x10^-11^). Only eight patients returned after the post-operative questionnaire to undergo additional imaging. All eight (100%) patients showed solid bony fusion as evident by blunting of screw threads and higher density within its lumen, without demineralization around the implant (Figure 6).

Effectiveness of fusion for increasing function

Patients were asked if they were satisfied with the procedure (Yes/No) and their improvement in pain and functionality; 94.7% (18/19) reported being satisfied with the procedure and experienced improvement in their pain and quality of life. They also reported improvement in function such as increased activity, ability to perform household chores, increased exercise tolerance, and better family interactions (playing with grandchildren). Overall, 16/19 patients reported improvement in their walking after the surgery, such as experiencing less pain while walking or being able to walk further. Of the three patients without improvement in walking, two reported the ability to stand longer (Table 3).

Statistical analysis of the data

A sample of 19 patients with back pain were analyzed before and after surgery. A numeric pain rating scale (NPRS) was used to measure pain, with 0 being no pain and 10 being maximum pain. The following hypotheses were tested:

Hypothesis 1: A prior lumbar fusion will lead to a smaller reduction in pain (prior lumbar fusion).

Hypothesis 2: A higher degree of pain reduction will occur after 12 months (the time after surgery).

 Hypothesis 3: Pain reduction will be smaller in older people (age 60 years or older) than in younger people (age 60 years or younger) (patients age).

For testing the hypothesis, “t-test: two-sample assuming unequal variance” was conducted using Microsoft Excel, where results were considered significant at a p-value of <0.05. There was no significant difference in pain reduction between the groups with or without prior lumbar surgery (t_12_ = 1.28, p-value = 0.22405). The pain reduction was not been significantly different before and after 12 months after surgery (t_14_ = 0.2539, p-value = 0.80322). Pain reduction was found to be unrelated to age (t_8_ = 0.1256, p-value = 0.90309), showing no significant difference between the ages of the groups. By proving the above hypothesis, the procedure is equally effective in treating people with or without prior lumbar surgery, everyone gets pain relief within the first year of surgery, and everyone experiences similar pain relief regardless of age.

## Discussion

The SIJ is a common source of low back pain, with some studies implicating the joint in 15 to 30% of cases of low back pain [1,2]. This large prevalence has led to an increase in surgical fusion of the SIJ to treat low back pain. Success of an SIJ fusion procedure can be defined objectively by radiographic fusion of the SIJ or subjectively by patient satisfaction and reduction in low back pain. Success is highly variable in the reported literature. One reason could be due to the difficulty of diagnosing SIJ pain [14]. Currently, there are no set diagnostic criteria. Diagnosis is often made through a combination of physical examination findings, imaging, and response to SIJ block injection, with SIJ block injection being the most predictive of SIJ fusion success [9,10,14].

In this series, successful pain reduction (> 50%) and improved functionality were seen in patients with positive and negative responses to SIJ injection with corticosteroids and local anesthetic block. The only patient with an unsuccessful outcome had a positive response to the injection. The two patients that had negative responses to SIJ block injection, and the patients who did not receive the injection all experienced a dramatic decrease in their pain following the procedure. These patients perhaps echo prior data showing the suboptimal sensitivity and specificity and as such reinforce the need to use radiographic and physical examination findings in conjunction with the SIJ block injection [14].

One patient in this series was not satisfied with the procedure, reporting a post-procedure pain score of 4. On the other hand, the other 18 cases were satisfied with the procedure despite residual pain, with post-procedure pain score of 5 in patient 14 and a score of 4 in patients 10 and 7. Interestingly, the unsatisfied patient had notable difference in preoperative pain, 5 compared to the cohort mean of 8.95 + 0.55, 95% CI, and percentage improvement in the unsatisfied was 20% compared to 50-100% in the cohort. This could indicate that patients with low pain scores are less likely to benefit from the procedure.

The Sacrix system uses screw implants imbedded with bone graft to provide immediate strong fixation across the joint, decrease future movement of the implant, and enhance fusion of the joint. The high rates of pain reduction following the surgery indicate good immediate fixation. In this series, post-operative radiological study showed solid fixation in all eight patients.

With the posterior oblique approach described in this paper, the screw entry point is on the upper outer surface of the iliac crest. This negates the need to dissect through the gluteal fascia to reach the ileum. This decreases the risk of injury to the superior gluteal neurovascular structures and the cluneal nerves. It also makes it a relatively bloodless procedure, which can be performed in an outpatient setting. This is in contrast to the lateral approach in which the average blood loss has been reported to be between 31 and 42.8 cc, and with an average length of stay between 0.8 and 1.9 days [15,16]. The posterior oblique approach also allows the surgical trajectory to remain within the ileum and sacrum, resulting in minimal soft tissue manipulation. Furthermore, the obliquity of the screw placement theoretically makes it less likely to distract compared to straight lateral approach in which the screws are end-on to the distraction forces. In a pilot study [17] including three patients using Sacrix, a lateral oblique approach is described with good results. The approach described in this paper is very similar to theirs. However, it is called posterior oblique because the insertion point of the screw is on the upper surface of the iliac crest and not on the lateral surface of the ilium.

Transloc (Cornerloc, Tulsa, OK) is another system similar to Sacrix. The authors were unable to find any journal article on it, probably because it is a new system. However, based on their brochure, they use a similar approach as Sacrix. Rialto (Medtronic’s, Minneapolis, MN) is another system that used similar technique to Sacrix. This system has shown to be safe and effective [18].

In the straight posterior approach in which a bone plug is inserted into the joint, there is distraction of the joint [19]. This is in contrast to the posterior oblique approach, in which the screws decrease movement in the joint by bringing the joint surfaces together. Theoretically, bringing the joint surfaces together should be more effective in decreasing pain because there is less room for movement within the joint. In addition, in the posterior approach, because the joint space is expanded, the bone graft may move, if fusion does not occur.

As the use of SIJ fusion increases, the number of newer systems will continue to increase. In a study by Himstead et al., in the last 10 years, there have been a total of 33 new devices in the market. The lateral approach had 21, posterior had six, and combination of posterior and lateral had six [20].

This study has limitations because the median for follow-up was only 12 months and the number of patients was small. Further studies with a larger number of patients and longer follow-up should be considered.

## Conclusions

Though this is a small series with a median follow-up of only one year, the results are very encouraging, with a high rate of pain reduction, increased functionality, and favorable post-operative radiographic findings. Furthermore, it is a minimally invasive and can be performed in an outpatient setting. Therefore, further studies on a larger number of patients with longer follow-up may be worthwhile.
